# Supplementary material for: Are carbon emissions trading and green financial instruments synergistic? -Comprehensive quantitative research based on content analysis
Source: PLoS One. 2024 Mar 7;19(3):e0298601. doi: 10.1371/journal.pone.0298601 (PMC10919601; doi:10.1371/journal.pone.0298601)
Supplement: S1 Data — (ZIP) [file pone.0298601.s001.zip › Data/地级市Z_F工作报告.zip!/║■─╧/╧μ╠╢╩╨/└┤╫╘╣1⁄2╓┌║┼í╛┬φ┐╦╩2╛▌═°í┐_2018.docx]

各位代表：

现在，我代表市人民政府向大会报告工作，请予审查，并请各位政协委员和其他列席人员提出意见。

一、2017年工作回顾

2017年是党的十九大胜利召开之年，也是本届政府履职的第一年。我们在省委、省政府和市委的坚强领导下，按照全年工作总体布局，砥砺奋进，乘势而上，全力以赴稳增长、积极稳妥促改革、久久为功调结构、精准施策惠民生、多措并举防风险，较好完成了市第十五届人民代表大会第一次会议确定的各项目标任务。

（一）坚持稳中求进总基调，发展步伐更加稳健。全年地区生产总值达到2055.8亿元，比上年增长8.3%。完成固定资产投资2204.9亿元，增长13.8%。完成财政总收入201.5亿元，增长8%；税收占比达到71.9%，比上年提高6.4个百分点。社会消费品零售总额645亿元，增长10.8%。进出口总额28.5亿美元，增长31.7%。引进内资341亿元，增长20%。利用外资12.12亿美元，增长14.9%。实现规模工业增加值917.1亿元，增长7.4%；高新技术产业增加值656.2亿元，增长13%。城镇居民人均可支配收入增长8.1%，达3.41万元；农村居民人均可支配收入增长7.9%，达1.79万元。居民消费价格指数控制在3%以下。万元GDP能耗下降3.7%。圆满实现年初确定的“两个2000亿、一个200亿”目标，特别是人均GDP突破1万美元大关，标志着湘潭的发展跃上了一个全新的台阶，湘潭人民逐梦前行的步伐更加稳健，更有力量。

（二）打好转型升级持久战，产业动能更为强劲。大力实施创新引领开放崛起战略，积极推进国家自主创新示范区、“中国制造2025”试点示范城市群、老工业城市产业转型升级示范区等平台建设，统筹实施制造强市、智能化改造、人才引进、产业招商等系列行动计划，形成了比较健全的工作机制和务实管用的政策体系。两大千亿计划全面发力，装备制造业产值达到913.3亿元，汽车产业产值达到296.4亿元，“1+4”特色产业占全市规模工业增加值达到了71.4%。湘钢实现工业总产值326亿元，吉利汽车实现工业总产值170亿元，湘潭经开区技工贸总收入突破2000亿元，成为全省第三个2000亿园区，重点产业、骨干企业的主导地位更加突出。狠抓产业项目的招商和开工，2016年招商签约项目开工率达到84%，2017年“港洽周”“湘商大会”等5大招商活动签约产业项目40个，已开工19个。湖南健康产业园核心区落户昭山。青山桥工业园获批省皮鞋皮具特色产业园。引进华夏幸福基业建设雨湖产业新城，在全省首开PPP模式整体开发运营产业新城的先河。以智造谷建设为总揽，新增16个国省创新平台、7个高端创新团队、5家省级智能制造试点示范企业及车间，机器换人和智能化改造取得新进展。湘潭高新区、天易经开区分获国、省双创示范基地，九华创新创业服务平台获批国家级科技企业孵化器，全社会研发经费占GDP比重达到2.1%。编制完成军民融合示范区建设规划。全国质量强市示范城市通过预验收。湘潭综保区获批筹建进口肉类指定查验场。成功举办中南农机展交会、金阳水果博览会、第十四届中国杜鹃花展、动漫嘉年华暨首届旅游节、首届芒果音乐节等系列会展旅游活动。

（三）落实城市发展新理念，城市建设更重内涵。《湘潭市城市总体规划(2010-2020年)》修订获国务院批准，编制完成教育、医疗等5项专项规划和九华等9项片区规划。全面加强城市设计与风貌管理，淡雅有致的城市风貌渐入人心。建成了熙春北路、江南大道、文星门路西段，提质了羊牯大道、广云东路等重要通道，拉通了河西城区段、易俗河段湘江防洪景观带，打通了三座大桥河西桥头、砂子岭等交通堵点。完成了建设路、韶山路、河东大道、双拥路等城市主干道“三化两拆两改”。窑湾、万楼等“千里湘江第一湾”项目初显雏形。仰天湖、碧泉湖、百亩湖等市民公园建设基本完工。莲城大道、芙蓉大道全面完成高质量硬化、高标准绿化、高品质亮化，以崭新的面貌呈现在世人面前，为莲城增添了两道靓丽的风景线。推进重点棚户片区和城中村片区改造，完成棚户区改造1.45万户。实施《湘潭市城市市容和环境卫生管理条例》，持续开展市容环境“六项整治”，城区主次干道违规招牌基本拆除，楼宇屋顶违法广告实现清零，亮化美化效果明显，城市面貌更加整洁清爽。新建和改造了一批社区公园、特色候车亭、环保公厕和垃圾中转站，启动9个立体停车场建设，完成40多处拆墙透绿。提质改造81个老旧小区，拆除了违章建筑，消除了照明暗区，实现了物业覆盖，市民生活更方便、更舒适、更惬意。湘潭迈入了颜值更高、品质更佳的发展阶段，城市向心力、美誉度大为提升。

（四）紧扣城乡统筹总抓手，农业农村更有活力。创新农业农村投融资机制，打造涉农资金整合的梅林桥模式，组建农业产业发展基金，现代农业综合示范园累计投资超过30亿元。加快农业供给侧结构性改革，农林牧渔业增加值达到159.3亿元，槟榔产业总产值突破150亿元，九华红菜苔、湘潭矮脚白获批中国地理标志产品，水府火焙鱼获中国中部（湖南）农博会金奖。落实粮食安全责任制，确保了区域粮食安全。推进农村经营体制改革，基本完成农村土地承包经营权确权登记颁证，新增耕地流转面积7.5万亩，流转率达到53%，特色产业园、示范合作社、家庭农场、乡村旅游点蓬勃发展。加快城乡基础设施建设，水府大桥合龙，水府大道全线拉通，韶乌线、莲花线、湘莲大道等干线公路建设如期推进，拓宽和硬化农村公路705公里，启动实施城乡统筹供水项目，完成8610处水毁水利工程修复。创建国家森林城市全面铺开，完成寻笔港退耕还林还湿项目。农村违章建筑和破败空心房专项整治有序推进，交通干线两厢绿化、美丽屋场建设成效明显，第二轮农村环境卫生综合整治全面完成。金石镇文星村等4个村镇获全国文明村镇，韶山市银田村获全国环境整治示范村、黄田村获中国美丽乡村百佳村，昭山七星村获中国十大乡建优秀探索奖，花石镇入选第二批全国特色小镇，美丽乡村的图景正在徐徐展开。

（五）把握深化改革主旋律，发展要素更显活跃。经济社会发展领域的105项改革任务顺利推进。深化“放管服”改革，市本级新取消26项行政审批项目，成立湘潭经开区行政审批局，赋予市级审批事项82项。全面落实商事制度改革，新增各类市场主体2.7万户，比上年增长45.4%。基本完成承担行政职能事业单位改革试点任务，市本级事业机构减少20个，划转行政职能2226项。推进金融改革，成立了市级资产管理公司，设立了中小微企业贷款风险缓释基金，市企业融资担保公司资本金增至25亿元。深化国有企业改革，稳步推进湘钢、湘电、江麓等驻潭国企“三供一业”分离，帮助企业聚焦主业加快发展。启动了政府投融资公司的清理、整合与转型发展，成立了市文化产业投资公司。推进财税制度改革，完善市本级财政全口径预算管理，实现国库集中支付乡镇街道全覆盖。推出政府性债务风险管控系列措施，市本级政府综合债务率下降了5个百分点。落实减税政策，全年减税约8亿元。推进国土改革，加强土地精细化管理，办理不动产登记业务23.5万宗。推进湘潭经开区增量配电改革试点，成立了全省首家配售电公司。深化人力资源改革，推出安家补助、创业扶持等12项措施，高校毕业生留潭率提高了4个百分点。加强社会信用体系建设，在全省率先对失信企业实施联合惩戒。随着各项改革的全面推进，资金、土地、人才等生产要素的发展活力和创新活力，在湘潭这片红色土地上竞相迸发。

（六）聚焦人民群众身边事，民生事业更具温度。稳步推进脱贫攻坚，13708人成功实现脱贫，农村贫困发生率降至1.39%。在全省率先建立稳定脱贫长效机制，扎实推进扶贫“六件实事”。投放小额扶贫贷款1.4亿元，完成207户易地扶贫搬迁，解决了5万多贫困人口饮水安全问题，完成危房改造3096户。农村电网改造计划进入第二年，解决了6053户村民“低电压”问题。新增城镇就业5.6万人、农村劳动力转移就业1.2万人。稳步提高农村低保、企业退休人员基本养老金和特困人员供养标准，城市社区全面建成老年人照料中心。完成市城区中小学校布局规划，新建公办幼儿园7所，改扩建义务教育薄弱学校99所，新增城区优质学位1.07万个，长郡高新实验学校、长沙市一中九华分校、风车坪建元学校等建成招生。获评全国未成年人思想道德建设工作先进城市，承办了省第二届中学生运动会，韶山学校、金庭学校获第一届全国文明校园称号。整合城乡居民医保，积极落实分级诊疗和医联体建设，公立医院综合改革取得阶段性成效，公共卫生服务稳步改善，重大传染病防控成效显著。荣获全国创建幸福家庭活动示范市。承办了首次国际A级足球赛事，电视剧《彭德怀元帅》荣获全国“五个一工程奖”，广播“村村响”实现全覆盖，“欢乐潇湘大美湘潭”“社区文明大舞台”等群众性文体活动广受欢迎。加强食药监管责任网格化、检查格式化、管理痕迹化，提质改造小餐饮199家、生鲜超市23个。严格落实安全生产责任制，事故起数和死亡人数实现“双下降”。加快建设领域历史遗留问题处置，妥善解决了16个开发合同遗留问题，为1.1万户办好了不动产权证。开展信访问题源头治理和积案清零行动，推进网上信访，一批信访问题得到化解。健全社会风险预警防范机制，社会治理精细化、智能化水平明显提高。强化社会治安防范，深化平安创建，刑事治安案件大幅下降，人民群众安全感持续提升，韶山市获全国社会治安综合治理最高奖“长安杯”。圆满完成村（居）换届选举。全面完成省9件12项、市10件27项民生实事，兑现了对全市人民的庄严承诺。

（七）始终坚持党的领导，自身建设更见成效。坚持在市委领导下积极主动地开展工作，落实市委的决策部署，重大事项及时报告。以迎接、学习、宣传、贯彻党的十九大作为首要政治任务，全面落实政府党组管党治党主体责任，扎实开展党建领航、“连心惠民”、主题党日等活动，“两学一做”学习教育实现常态化、制度化。落实党风廉政建设“两个责任”，审计监督与行政监察力度进一步加大。坚持政府全会、政府常务会、市长办公周例会等议事决策机制，落实重点任务市长交办函等督查督办制度，推进政府工作的规范高效运转。实施市级领导领办重点产业项目、对口帮扶企业、联系棚户区改造等中心工作。坚持依法科学民主决策，推行政府顾问、法律顾问制度，全面落实重大决策合法性审查和“三重一大”制度。12345市长热线、市长信箱全年接收群众来信来电近6万件，办结率达到98%，确保了民意诉求在第一时间得到处理。坚持勤俭办事，“三公经费”同比下降20%左右。落实重大事项向市人大及其常委会报告和向市政协通报制度，认真执行市人大及其常委会决议、决定，自觉接受人大、政协和社会各界监督，办理人大代表建议和政协提案852件，办结率和满意率均为100%。民族宗教、妇女儿童、青少年、老龄、工会、科协、社科联、侨联、文联、残联、韶灌、人防、移民、气象、水文、国安、档案、市志、公积金、供销合作等工作取得新进展。综治工作、计划生育、安全生产、打非处非等多项工作获评全省先进或优秀等次。

市十五届人大一次会议上，政府工作报告部署安排了159项重点任务，有154项基本完成或达到进度要求。未达到预期进度的工作有河东桥头交通优化和立体停车场建设。未达到预期目标的指标有3项，固定资产投资增速与目标值相差1.2个百分点，社会消费品零售总额增速相差约1.2个百分点，农村居民人均可支配收入增速相差0.6个百分点。这些问题，既有客观因素的制约，也反映了我们工作和发展中存在的不足。

各位代表，2017年，在市委的坚强领导下，我们在统筹推进各项工作的同时，突出重点、集中力量办成了几件大事。

一是成功创建全国文明城市。2017年是十四载创文接力赛夺标冲刺之年。一年来，我们紧紧依靠全市人民，狠抓各项基础建设、营造良好公共秩序、培育文明社会风尚，跨越一个个台阶，解决一个个难题，发起一轮轮攻坚，全市上下携手并肩、戮力同心，共同托起了湘潭的“创文梦”。湘潭市、韶山市双双获评全国文明城市。多年夙愿，一朝梦圆。湘潭人民的自豪感、荣誉感和归属感全面增强，汇聚起了昂首阔步、擘画未来的磅礴之力。

二是战胜了特大暴雨洪涝灾害。面对灾情，我们始终把人民群众生命财产安全放在首位，各级干部以实际行动经受住了“入职大考”，党员干部、人民群众、子弟兵、民兵预备役与企业、社团、志愿者等社会各界栉风沐雨、同舟共济，实现了“不垮一库一坝、不溃一堤一垸、不发生群死群伤”的目标，夺得了抗洪抢险斗争的全面胜利，确保了社会稳定、民生安定、重建有力，铸就了“众志成城、百折不挠、顽强拼搏”的抗洪精神，展开了一幅幅壮阔的感人画卷。

三是取得了优化发展质量的良好开局。在全力以赴稳增长的同时，我们坚定不移地促进实体经济、提升发展质量。第三产业占比首次突破40%，工业投资占比达到49.1%，非公经济占规模工业产值达到83.2%，民间投资增速比上年提高了17.5个百分点，地方一般预算收入中非税收入占比较上年下降了6.6个百分点，产业结构、投资结构、收入结构明显优化，为更高质量、更有效率、更可持续、更加公平的发展奠定了坚实基础。

四是打响环境治理攻坚战。牢牢扛起生态文明建设的政治责任，层层落实责任清单，全力抓好中央环保督察问题整改。河长制全面推行，黄标车全部淘汰，水府庙网箱拦库全域退出，湘江、涟水采砂全线取缔，“一江两水”城区段137艘“三类船舶”整治清零，竹埠港化工企业整体拆除，完成城区18条黑臭水体治理和一水厂、三水厂水源地保护工程，在全省率先成立环境犯罪刑事侦查支队，“绿水青山就是金山银山”的发展理念在湘潭落地生根，生态文明建设翻开了崭新的篇章。

五是全面铺开精美湘潭项目建设。2017年是精美湘潭项目建设决战决胜的一年。我们既尽力而为，又量力而行，合理铺排项目，积极创新融资，多方论证方案，科学组织施工，实行了“五个同步”监管。重点棚户区改造、城际通道提质、城区交通优化、“三化两拆两改”次第铺开，相继见效。一年来，我们办成了一批群众期盼已久的大事，解决了一批市民长期关注的难事，湘潭这座城市呈现出日进日新的蓬勃之势。

各位代表，回顾2017年的不凡历程，我们深深地懂得，只要坚持以人民为中心，就能取得全市人民的支持，凝聚全市人民的力量，就能精进前行、驰而不息，稳步达到我们的目标。这些成绩的取得，离不开省委省政府和市委的坚强领导，得益于市人大、市政协的监督支持，是全市人民共同努力的结果。在此，我代表市人民政府，向各位代表、各位委员，向奋战在各条战线上的广大干部群众、各民主党派、工商联、人民团体，向离退休老同志和所有关心支持湘潭发展的朋友，向驻潭部队、公安民警和武警官兵，表示崇高的敬意和衷心的感谢！

在总结成绩的同时，我们必须清醒地认识到：新兴产业的主导地位、引领作用还不强，经济动能转换新不足以补旧、小不足以补大的矛盾比较突出；中小企业仍然较为困难，支持实体经济发展的力度有待增强；重大项目储备不足，投资接续能力不强，出口支撑作用有限，经济增长的基础还不牢固；要素供给能力有待提高，资金、人才的短板依然明显；环境问题历史欠账较多，大气污染防治任务艰巨，雾霾天气仍有发生，河湖水质还有反复，土壤污染整治任重道远；一些部门和干部依然存在习惯思维和路径依赖，思想不够解放，工作不够主动，担当担责的意识不强，“表态多调门高、行动少落实差”等形式主义、官僚主义新表现时有发生，公共服务水平与效率离企业、群众的期盼有较大差距。在今后的工作中，我们将坚持问题导向，下更大力气，想更多办法，努力解决这些问题。

二、2018年工作安排

2018年，是贯彻落实党的十九大精神的开局之年，是改革开放40周年，是决胜全面建成小康社会、实施“十三五”规划承上启下的关键一年，是开启湘潭基本现代化建设新征程的重要一年。从国家层面来看，落实党的十九大作出的重大战略部署，推动高质量发展将是今后一个时期的根本要求。从省内来看，长株潭一体化将按下快进键，省委省政府将以更大的力度打造长株潭核心增长极。从自身来看，经过长期努力，湘潭发展到了厚积薄发的关键时期，尤其是近两年来，湘潭人民实现了创建全国文明城市等多年梦想，全市上下凝聚了一股建设“伟人故里、大美湘潭”的强大合力。

各位代表，面对新机遇要有新作为，开启新征程要有新状态。新的一年，我们将以习近平新时代中国特色社会主义思想为指导，坚持稳中求进工作总基调，坚持新发展理念，按照高质量发展的要求，根据中央、省委、市委经济工作会议的部署，以供给侧结构性改革为主线，大力实施创新引领、开放崛起战略，推动经济社会持续健康发展。主要预期目标为：地区生产总值增长8.5%左右；固定资产投资增长12%以上；规模工业增加值增长8%左右；财政总收入增长7%以上，非税收入占地方收入比重控制在42.8%以下；社会消费品零售总额增长11%左右；进出口总额增长16%左右；引进内资增长14%左右，实际利用外资增长12%左右；城乡居民人均可支配收入增长8%以上。

为实现上述目标，我们将开展好五大主题年活动。

一是产业项目建设年。重点围绕“1+4”特色产业，持续推动装备制造产业千亿倍增计划和汽车产业千亿行动计划，启动军民融合产业千亿行动计划。全年引进500强企业15家，开工10亿元以上产业项目10个，重点产业项目投资突破800亿元；规模工业增加值突破1000亿元，规模工业企业突破1000家；装备制造产业产值突破1200亿元，汽车产业产值突破600亿元，军民融合产业产值达420亿元以上。

二是园区发展提质年。完善各园区产业规划，探索“一园一链”“多园一链”等特色化、统筹化园区发展模式，深入推进赋权强园，理顺园区管理体制，支持园区各项改革。完善园区考核办法，加强对工业产值、财政收入、投资等经济指标的考核，加大规模工业增加值、税收占比、产业投资、科技投入等质量指标的考核权重。力争全市80%以上的规模工业增加值、80%以上的高新技术产值、80%以上的财税收入来自园区，努力实现增速更稳、效益更好的高质量发展。

三是环境治理攻坚年。重点打赢蓝天保卫战，统筹推进碧水攻坚战和净土持久战，全年空气质量优良率达到78%以上，水环境质量稳定达标，万元GDP综合能耗下降3.6%以上，全面完成污染物排放总量削减省定目标，守住环境质量只能更好、不能变坏的责任底线。开展环保综合督查，强化环保综合执法，深化生态文明体制改革，加强生态文化建设，推进生态修复工程和全域绿化行动计划，努力形成保护、治理、修复并重的生态文明建设新局面。

四是精美湘潭深化年。切实巩固和深化精美湘潭建设成果，重点开发建设窑湾、万楼、宝塔、竹埠港四大片区，打造“一江两岸”城市主轴。深入开展国家新型城镇化综合试点，统筹推进城市双修、海绵城市、智慧城市、地下综合管廊、新一轮棚户区改造和城市管理标准化等各项工作，提升城市生活品质，实现更有内涵、更有质量的精明增长。深入创建国家卫生城市、国家森林城市、全国交通畅通模范城市，建设人民群众更加满意的全国文明城市。

五是行政效能优化年。以执行力建设为主线，开展政务大督查，全面落实依责承诺、尽责践诺、问责督诺、全责履诺。深化“放管服”改革，积极推进多评合一、多规合一、多图联审和行政许可标准化，完善智慧政务平台，建立“马上就办”和“最多跑一次”工作机制，行政审批提速率达15%以上。有效解决“脸好看、事难办”“不贪不占也不干”等“四风”问题，着力为就业创业降门槛，为市场主体减负担，为激发投资拓空间，为公平营商创条件，为群众办事增便利。

重点抓好以下十个方面的工作：

（一）抓优势产业，加快形成先进制造业集群。突出“1+4”特色产业，更好发挥智能制造等企业联盟作用，培育壮大海工装备等新兴优势产业链，推行核心企业担任“盟长”、市级领导担任“链长”的配套协作机制。全力保持湘钢、吉利汽车、泰富重装、桑顿新能源等骨干企业的发展势头，加快吉利新能源SUV、新松机器人、F2国际赛道、众泰、舍弗勒、屹丰、蓝思科技等重点项目建设，努力挖掘湘电、威胜等企业增长潜力。加大胜利钢管、地通汽车、北汽大世等中小企业扶持力度，推进中小企业入规和上台阶。严格兑现企业引进产业链和本地采购系列奖励措施，推进核心企业订单融资、应收账款融资，有效提升本地配套率，推动引链补链强链。鼓励企业发展直接融资，启动企业上市“蝶变”计划，重点支持10家企业加快主板上市步伐，鼓励中小企业股改，培育上市企业后备梯队。编制军工城建设专项规划，以湘电、江麓、江南、开启时代、崇德科技等企业为依托，推进军民深度融合，积极创建国家级军民融合创新示范区。深入实施质量强市战略，创成全国质量强市示范城市。支持湘潭高新区构建“3+1”产业体系，建设智造谷核心区，实现争位进位。支持湘潭经开区建设汽车产业城，工业总产值突破1500亿元，财政总收入突破55亿元。加快华夏幸福雨湖产业新城建设，完成管理机构搭建和规划编制，开工建设展示中心、产业港、主干道等先导项目。

（二）抓创新引领，围绕产业链部署创新链。深入推进以科技创新为核心的全面创新，争创国家创新型城市。深化智造谷建设，加快智能化改造专项行动，继续推进机器换人和智能化改造项目，培育一批智能制造试点示范企业和车间，积极申报“中国制造2025”国家级示范区。聚焦自主创新示范区标志性创新项目，依托优势产业，重点培育10大产业集群创新链、10大科技创新龙头企业、10大科技创新项目、10大知识产权优势企业，形成产业创新发展的骨干力量。力争全社会研发经费投入强度达到2.2%，新增高新技术企业60家以上，总数突破180家，科技进步贡献率达到54%，推进湘潭制造业迈向中高端。加强企业孵化平台、协同创新平台、国省双创示范园区等平台建设，加快院士创新产业园、力合科创领航城发展。加快文化创新步伐，加强对红色文化、湖湘文化、白石文化、军工文化等文化资源的挖掘、提升与推广，举办第五届中国（湘潭）齐白石国际文化艺术节，着力培育文化企业、塑造文化品牌。落实产业人才系列政策，强化引进人才的硬政策，优化留住人才的软环境。努力将高等院校毕业生留潭率提高5个百分点，留潭人数达到5000人以上。持续推进校地深度融合和产学研合作，全力支持湘潭大学、湖南科技大学等驻潭高校和职业院校发展，推动吉利学院、软件学院专升本，努力培育具有创新意识的科技人才队伍和具备工匠精神的技工人才队伍。打造聚才用才高地，厚植创业创新沃土，让创新之花在莲城大地精彩绽放。

（三）抓开放崛起，不断拓宽发展空间。立足长株潭核心增长极，落实“五个对接”行动，加快形成全面开放新格局。聚焦世界500强、中国500强、民营企业500强，抓好招商引资项目库和客户资源库建设。紧盯长三角、珠三角、京津冀，办好装备制造、文化旅游、健康养老等招商推介会，举办首届潭商大会，组织参加“沪洽周”等招商活动，在招大引强上考规模，在项目落地上考进度，在开工建设上考实物，在投产达效上考产值。积极组织企业参与东盟博览会、亚欧博览会、汉诺威工业博览会等会展活动，支持企业参与“一带一路”建设。推进综保区和经开区一体化发展，加快建设综保区二期。大力发展商贸物流业，确保金阳商贸城二期、岳塘国际商贸城一期建成开业，支持岳塘经开区打造长株潭中央商贸区。依托区位优势和“互联网+”，衍生跨界融合、智能营销等新模式，加快京东项目的落地建设，支持步步高发展智慧零售，努力打造智慧物流2.0时代的长株潭物流中心和中南地区物流枢纽。加快湖南健康产业园核心区建设，推进恒大健康、北大未名等项目，支持昭山创建国家级文化产业示范园区。深入创建全域旅游示范城市，支持彭德怀纪念园、盘龙大观园创建国家5A级景区，支持韶山、昭山创成国家全域旅游示范区，策划好窑湾景区开街系列活动，打造湘潭旅游新名片。结合“不忘初心、牢记使命”主题教育，推进韶山、乌石等红色旅游教育基地建设。努力引进国际知名品牌酒店，切实提升旅游接待能力。办好湖南红色旅游文化节、春季乡村旅游节、中国湘菜美食文化节等节会活动，不断提升热度、做旺人气。

（四）抓县域经济，构建竞相发展新格局。完善全市县域发展规划布局，启动县域经济发展五年行动计划，研究出台支持县域经济发展的系列政策。向县一级下放更多市级权力，支持县级开展行政权力集中审批改革，努力实现审批权力闭环运行。着力破解县域发展用地难题，强化项目用地保障。支持湘潭经开区、高新区等国省级园区领办、共建县域产业园区，鼓励县域引入战略投资者采用PPP模式推进片区开发，打造金石等一批特色产业园。加快建设韶乌线、伏林大道、G320绕城线，拉通县域快速通道。出台县域经济发展考核奖励办法，对在全国县域经济排名中实现进位赶超的县市，加大政策支持和资金奖励力度。支持湘潭县创建国家一二三产业融合发展先导区，开发建设杨河片区，启动五年迈进“百强县”计划；支持天易经开区发展装备制造、食品医药、新材料三大主导产业和槟榔、湘莲、海泡石三大特色产业，进军“千亿园区”，争创国家级园区。支持湘乡市推进老工业基地转型升级，加快迈向“百强县”；支持湘乡经开区打造电工电器、机械制造、环保电镀3个百亿产业集群；支持水府示范区提升景区功能，争创国家级旅游度假区。支持湘潭县、湘乡市开展全国文明城市创建活动。支持韶山市加快核心景区提质工程、“最忆韶山冲”文旅综合体、韶山干部学院等项目建设，着力打造红色教育培训产业，建设世界知名旅游目的地；支持韶山高新区发展食品、新材料及高端制造业。继续实施乡镇经济上台阶奖励政策，推进经济较发达乡镇扩权强镇试点，支持花石、棋梓、银田等重点镇、特色镇基础设施建设，实现“一镇一业、一镇一品”特色发展。努力构建园区经济、县域经济、乡镇经济竞相发力的新格局，汇聚湘潭新时代发展的源头活水、滚滚洪流。

（五）抓环境治理，全力推进绿色发展。着力打好蓝天保卫战，实施大气污染防治行动计划，继续采取“五个一律”断然措施，全面整治企业排放、工地扬尘、秸秆焚烧、餐饮油烟、汽车尾气等污染源，力争空气优良天数稳步增加，空气质量指数持续好转。以河长制为抓手，深入推进湘江保护与治理第二个三年行动计划，进一步加强饮用水源地环境保护，全面完成城区黑臭水体基础治理，确保河流水质稳定达标、县级以上集中式饮用水100%达标。强化农业面源污染防治，加快农村垃圾处理体系建设和禁养区内畜禽规模养殖场关闭退出，推进病死畜禽无害化处理中心建设。加大重金属污染耕地修复治理力度，打造竹埠港土壤修复样板工程。严守生态保护红线，完善绿心地区规划，稳步建立限开区、禁开区生态保护和补偿机制。全面开展污染源普查，有力提升环保执法水平，严厉打击破坏生态、污染环境的违法犯罪行为。推行领导干部自然资源资产离任审计，落实领导干部生态环境损害责任追究办法。建成锰矿国家矿山公园，建设东台山、白石、仙女山、褒忠山等国省级森林公园。启动百里森林廊道和城市森林廊道建设，推进城市复绿、透绿和立体绿化。坚持自然恢复为主，推进湘江、涟水、涓水两岸生态修复工程，沿一江两岸布局田园综合体、绿道、自然生态驳岸、退耕还湿等生态项目，让两岸苍翠守护一江绿水，让一江清水滋养湘潭大地。

（六）抓城市品质，建设更加精美的宜居家园。始终坚持在长株潭城市群的大格局中谋划湘潭的城市建设和发展，稳步推进城际轨道等重大交通基础设施项目，加快实现昭山路、湘江风光带等城际干道互联互通。严格执行新版城市总规，实施历史建筑和历史文化街区保护条例，打造精致精美的城市气质与特色风貌。围绕“一江两岸五山十八总”，对标5A级景区标准，加快建设“千里湘江第一湾”。实现窑湾片区历史文化街区一期开街运营，加快万楼片区“一馆五中心”等基础设施建设，有序推进宝塔片区整体开发，实现竹埠港片区招商引资和基础设施建设新突破，全线贯通湘江两岸防洪景观带，集中力量打造城市主轴。启动新一轮棚户区改造三年计划，年内完成1万户改造任务，加快窑湾、河西滨江、板塘、竹埠港等棚户片区清零。加快建立多主体供应、多渠道保障、租购并举的住房制度，完善促进房地产市场平稳健康发展的长效机制。推进莲花线、电工北路、潭锰路建设，加快打造建设路南北大走廊。努力打通西南二环与东北二环，完善城市骨架。建成昭华大桥，加快杨梅洲大桥、下摄司大桥建设，建好荷塘客运枢纽站、河西公交客运总站。有序推进城市双修，启动地下管网普查，开展雨污分流、供水老旧管网、城市积水点改造，建好赤马路、涟滨路等地下综合管廊，完成过江桥梁“桥头堡”建设和交通优化。加大拆违控违和征地拆迁“清零”力度，编制和实施城市美化绿化亮化标准规范。建设一批公园、停车场、人行通道、公共厕所、公交车站等便民设施，继续推进老旧小区改造和垃圾分类处理设施建设，让城市生活更加和谐宜居、更加幸福美好。

（七）抓乡村振兴，推进农业农村优先发展。打好精准脱贫攻坚战，完成年度减贫任务，实施省定贫困村振兴工程，落实稳定脱贫长效机制。以国家现代农业示范区建设为抓手，加快农业供给侧结构性改革，建设梅林桥等十大现代农业综合示范园，推动一二三产业融合发展。全力打造槟榔、湘莲、沙子岭猪等“潭字号”特色农产品品牌，加快培育休闲农业、乡村旅游、智慧农业、农村电商等新模式新业态。支持龙头企业和农民合作社、家庭农场等新型经营主体发展壮大。实施优质粮油工程，大力发展粮食产业经济，确保“米袋子”“菜篮子”优质安全供应。启动实施“三乡”工程，大力推进能人回乡、产业兴乡、市民下乡，鼓励企业家、能人到乡村投资兴业，引导市民租用农村空房空地发展乡村旅游、居家养老等产业，推动资源、资金、人才回流农村，振兴乡村。实施农村双改，努力实现全市村庄规划全覆盖，探索以组为单位的村民集中居住试点，推进农村人居环境综合整治。打造美丽乡村升级版，加快建设花石湘莲小镇等特色小镇和韶山平里等田园综合体。建好、管好、护好、运营好农村公路。有序推进水毁水损工程修复、水土保持和生态小流域治理、引涟济潭、城乡统筹供水、光纤入村等项目建设。完成电网改造三年行动计划，全面解决农村“低电压”问题。启动农村集体产权制度改革，深化土地“三权分置”改革，开展土地流转履约保险试点。支持村级集体经济发展，抓好财政专项支持村级集体经济发展试点。进一步完善村务公开、村组财务管理等机制，稳妥推进并村后续工作。发展农业信贷担保，支持“一城一行、一县两行”发展。完善现代农业社会化服务体系，深化供销合作社综合改革，加大科技兴农力度。加强基层基础工作，健全自治、法治、德治相结合的乡村治理体系。

（八）抓民生事业，切实增强人民群众获得感。推进教育强市三年行动计划，完成滴水湖小学、湘乡起凤学校等4所学校建设，启动建设湖湘小学、万楼中学。建设教师发展中心，加强师资力量培养，进一步提高教师队伍整体水平。加快义务教育一体化改革，启动国家义务教育优质均衡县（市区）创建，积极建设全国研学旅行试验区。适应高考改革新形势，推进高中阶段教育创新。加强幼儿教育，发展普惠性幼儿园。加强全民国防教育，支持国防建设和军队改革。深化医药卫生体制综合改革，推进药品带量采购、按病种收付费等改革，提升基层医疗卫生机构服务能力。推进医联体建设，促进分级诊疗。广泛开展爱国卫生运动，积极发展中医药事业，加快市中心医院九华分院、市中医院建设。深入实施文化惠民工程，推广全民健身运动，加快市民之家、市体育中心、全民健身中心等阵地建设，推进城乡文体场地设施广覆盖。加强对高等院校毕业生、返乡农民工等重点人群带头创业的政策扶持力度，注重解决就业结构性矛盾，扶持一批就业安置基地和创业孵化基地。加强人口计划生育工作，积极应对老龄化，打造湘潭养老服务品牌。稳步增加企业退休职工养老生活补助和养老金，稳定提升城乡低保、最低工资、基本医疗保险补助、特困人员供养等社会保障标准，完善医疗救助、急难救助、临时救助和慈善援助为主体的贫困救助系统。全力办好厕所革命、四好农村公路、老旧小区改造等10件民生实事，把一桩桩好事、一件件实事办到人民群众的心坎上。

（九）抓风险防范，确保社会大局和谐稳定。严格执行国家有关政策，全面落实政府性债务管理系列规定，持续规范债务举借、使用、偿还、风险预警等流程，积极稳妥置换和化解存量债务，市本级力争消化政府性债务余额5%以上，将政府综合债务率控制在安全区内。以市场化、实体化为导向，推动政府投融资公司转型发展，努力实现资金筹措、项目运营、融资偿还的良性运转。切实防止金融脱实向虚，促进金融服务实体经济，加大对中小微企业的融资支持。加强地方金融类机构监管，加大对互联网金融、民间融资的风险排查，强化信贷资产质量日常监测与评估，坚决依法打击失信行为，保持良好金融生态。加快处置僵尸企业，释放更多土地和资产。以创建国家安全发展示范城市为抓手,强化监管执法和企业主体责任，加强安全网格化管理、重点行业专项整治和重点区域监管，全力防范遏制各类事故发生。加快综治中心、雪亮工程和网格化服务管理“三位一体”建设，推进平安湘潭创建常态化。全面开展信访“三无”乡镇创建，积极化解信访积案。更加重视社会热点问题，加强网络舆情应对，强化网络信息安全。从严防控廉洁风险，紧紧看住和管好用好扶贫、低保、医保等民生资金，加强对农村集体资金、资产、资源监管。完善应急管理，高效稳妥应对重大自然灾害、地质灾害、疾病疫情和各类突发事件。以创建国家食品安全示范城市为总揽，健全食品安全追溯体系，建设智慧监管平台，全力保障人民群众“舌尖上的安全”。

（十）抓自身建设，打造人民群众更加满意的服务型政府。学习宣传贯彻好党的十九大精神，以习近平新时代中国特色社会主义思想武装头脑、指导实践、推动工作。始终坚持党的领导，严格落实向市委常委会报告工作、“三重一大”等制度，确保中央、省和市委的决策部署在政府系统不折不扣地得到落实。依法接受人大及其常委会监督，自觉接受政协民主监督，做好建议、提案办理工作。主动接受社会监督、舆论监督和司法监督，认真听取人大代表、政协委员、民主党派、工商联、人民团体和社会各界的意见。落实政府党组全面从严治党主体责任，支持监察体制改革。坚持用制度管权管事管人，着力防止“小范围、小活动、小表示、小变通”的“微腐败”。政府及其工作人员要惯于在阳光下工作生活，善于在聚光灯下履职尽责，敢于在显微镜下接受检视，始终保持风清气正、崇廉尚实的良好政风。打造一流的执行能力，把狠抓落实、强化执行作为鲜明导向，坚持以实绩衡量工作、用担当考量干部，只容为公之失，不赦为私之过，努力形成有决议必执行、有责任必担当、有任务必完成、有交办必回复的工作标准和办事规范。树立一流的法治观念，深入贯彻全面依法治国要求，加快推进政事分开、综合执法等改革，全面落实行政执法责任制，推行行政机关负责人出庭应诉，深化“互联网+监督”，严格规范执法行为，决不允许以言代法、以权压法、逐利违法、徇私枉法。锤炼一流的务实作风，不提脱离实际的高指标，不喊哗众取宠的空口号，不搞劳民伤财的假政绩，努力形成务实而不好高骛远、创新而不因循守旧、守恒而不朝令夕改、稳健而不急功近利、亲民而不高高在上的优良作风。推行一线工作法，真正做到问题在一线解决、矛盾在一线调处、局面在一线打开、成绩在一线检验。追求一流的工作成果，以扛得住嘘声、听得进骂声、经得起捧声的定力，拿出燕子垒窝的恒劲、蚂蚁啃骨的韧劲、老牛爬坡的拼劲，干成一批着眼长远的大事，攻克一批错综复杂的难事，办好一批群众关注的实事，努力交上一份让人民群众满意的答卷。

各位代表，我们身处“两个一百年”奋斗目标的历史交汇期，肩负奋力开启湘潭基本现代化建设新征程的重大使命。道虽迩，不行不至；事虽小，不为不成。让我们高举习近平新时代中国特色社会主义思想伟大旗帜，在省委省政府和市委的坚强领导下，为建设富裕湘潭、法治湘潭、文化湘潭、幸福湘潭、美丽湘潭而努力奋斗！
